# Analysis of the spatiotemporal dynamics of vascular injury and regeneration following experimental Spinal Cord Injury

**DOI:** 10.1016/j.bas.2025.104191

**Published:** 2025-01-23

**Authors:** Christian J. Entenmann, Emily J. von Bronewski, Lilly Waldmann, Lea Meyer, Katharina Kersting, Laurens T. Roolfs, Lasse M. Schleker, Melina Nieminen-Kelhä, Irina Kremenetskaia, Frank L. Heppner, Michael G. Fehlings, Peter Vajkoczy, Vanessa Hubertus

**Affiliations:** a Department of Neurosurgery, Charité – Universitätsmedizin Berlin, Corporate Member of Freie Universität Berlin, Humboldt-Universität zu Berlin, Berlin Institute of Health, Berlin, Germany; b German Center for Neurodegenerative Diseases (DZNE), Berlin, Germany; c Cluster of Excellence, NeuroCure, Berlin, Germany; d Division of Neurosurgery and Krembil Neuroscience Center, Toronto Western Hospital, University Health Network and University of Toronto, Toronto, Canada; e Berlin Institute of Health (BIH) – Charité Clinician Scientist Program, Berlin, Germany

**Keywords:** Spinal cord injury, Vascular injury, Vascular proliferation, Blood spinal cord barrier, Revascularization, Angiogenesis

## Abstract

**Introduction:**

The loss of vasculature in Spinal Cord Injury (SCI) contributes to secondary injury, expanding the injury to unharmed spinal cord (SC) regions. Understanding these mechanisms is crucial for developing therapeutic interventions.

**Research question:**

Comprehensive analysis of the temporospatial dynamics of vascular injury and regeneration following SCI.

**Materials and methods:**

Adult C57BL/6J mice were subjected to clip-compression SCI (Th 6/7, 5g, 60s, n = 20) or sham injury (laminectomy, n = 4), and sacrificed at 1, 3, 7, 14, and 28 days (d) post-injury following intracardial fluorescein isothiocyanate (FITC)-Lectin perfusion. Histological analysis (CD31, FITC-Lectin, Ki-67, IgG, TER-119) assessed vascular changes, permeability, and proliferation within the injury epicenter (region 0 (R0), ± 0,5 mm) and two adjacent SC regions (R1: ± 1 mm, R2: ± 2.5 mm).

**Results:**

Perfusion loss (FITC-Lectin+/CD31+), was most severe in R0 and R1 at d3 (p < 0.01). Significant vascular loss in R2 started at d3 (p = 0.043). Perfusion was restored at d28 in R0 and R1, and at d7 in R2. Vessel density (CD31^+^) returned to baseline quicker (R0: d3, R1 and R2: d14). Vascular proliferation (CD31+/Ki-67+) manifested across all regions at d3 (p < 0.01), and most notably in R2 (p < 0.01). Vascular permeability for IgG remained disrupted until d3 in R0 and R1 and until d14 in R2.

**Discussion and conclusion:**

Vascular injury is most severe initially and spreads to the surrounding SC regions. Gradual vascular regeneration occurs early and up to a considerable distance from the injury epicenter, highlighting the potential of early therapeutic interventions targeted at vascular repair and regeneration.

## Highlights

• Comprehensive analysis of the spatiotemporal dynamics of vascular injury and revascularization following Spinal Cord Injury.
• Rapid vascular density loss at the injury epicenter by day one with delayed perfusion loss spreading to surrounding areas.
• Vascular proliferation in distant regions surmounts regeneration in the injury epicenter, peaking after three days.
• The blood-spinal cord barrier is compromised early with a peak on day one at the epicenter and day three in distant regions.
• This study highlights the potential of early therapeutic interventions targeted at vascular repair and regeneration.

## Graphical abstract

**Figure:**
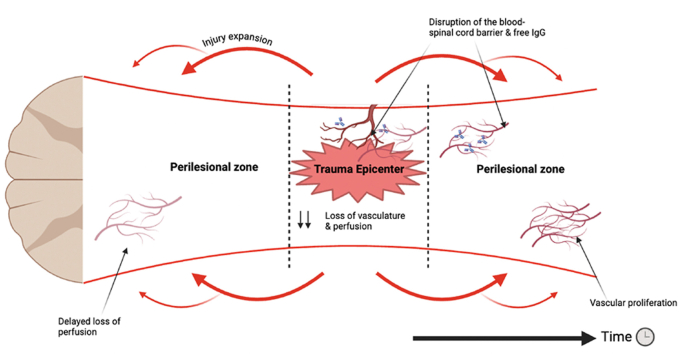

## 1 Introduction

Spinal Cord Injury (SCI) remains a leading cause of disability, imposing a significant burden on healthcare systems worldwide. Despite recent advancements in the medical, surgical and rehabilitation care for SCI, neuroregenerative approaches to restore impaired spinal cord (SC) function are lacking (Kwon et al., 2024), making SCI as relevant today as it was in the past (Ahuja et al., 2017a; Safdarian et al., 2023). In Europe, its yearly incidence is estimated at around 11,000 cases, with a prevalence of approximately 300,000 affected patients (Safdarian et al., 2023; Paraplegiologie DMGf; Rupp et al., 2021). Globally, these numbers have been steadily increasing over the past decades (Safdarian et al., 2023). Currently, there are estimates of a global prevalence of around 15–21 million affected patients and an annual incidence of 700.000–1.2 million new cases of SCI (Safdarian et al., 2023; Ding et al., 2022; Kumar et al., 2018; WHO, 2024).

Similar to other central nervous system (CNS) injuries, SCI is characterized by primary and secondary injury mechanisms. The primary injury results from the initial trauma, causing immediate damage to the SC vasculature and neural tissue (Ahuja et al., 2017a, 2017b; Ng et al., 2011; Tator, 1991, 1995; Tator and Fehlings, 1991). This is followed by a secondary injury cascade, where ongoing pathological processes lead to further vascular and neuronal damage (Ahuja et al., 2017a; Ng et al., 2011; Tator, 1991, 1995; Tator and Fehlings, 1991; Mautes et al., 2000). The ensuing post-traumatic ischemic environment exacerbates neural tissue loss and significantly impedes the regenerative capacity of the SC (Ahuja et al., 2017a; Tator and Fehlings, 1991). Endogenous attempts of the SC at vascular regeneration, including reperfusion and angiogenesis, primarily occur within the first week post-SCI and might be crucial for neural recovery and functional outcomes (Bearden and Segal, 2004; Ohab et al., 2006; Roolfs et al., 2022; Fassbender et al., 2011; Hubertus et al., 2024), as neurons and axons have been shown to grow along newly formed vessels (Bearden and Segal, 2004; Ohab et al., 2006). Notably, a second phase of pathologic permeability increase of the blood-spinal cord barrier (BSCB) occurs during this time, representing a potential therapeutic window for interventions aimed at enhancing recovery (Roolfs et al., 2022; Figley et al., 2014a; Loy et al., 2002).

Previous studies have explored different aspects of vascular injury and regeneration in parts before but have often been limited to narrow time frames - such as the hyperacute or acute phase after SCI (hours to a couple of days) - or have focused on singular aspects of vascular disruption like changes in BSCB permeability (Loy et al., 2002; Popovich et al., 1996; Noble and Wrathall, 1988, 1989; Cohen et al., 2009). We believe that a comprehensive understanding of vascular changes across multiple time points representing progressive phases in SCI, combined with the analysis of different regions of the SC, is crucial for developing effective therapies that target the vasculature. In this study, we employ a well-established clip contusion/compression murine model of experimental SCI to investigate vascular changes across multiple SC regions over the course of 28 days, thus providing a detailed immunohistochemical picture of the vascular responses of the SC after SCI.

## 2 Methods

### 2.1 Induction of spinal cord injury

**Fig. 1:**
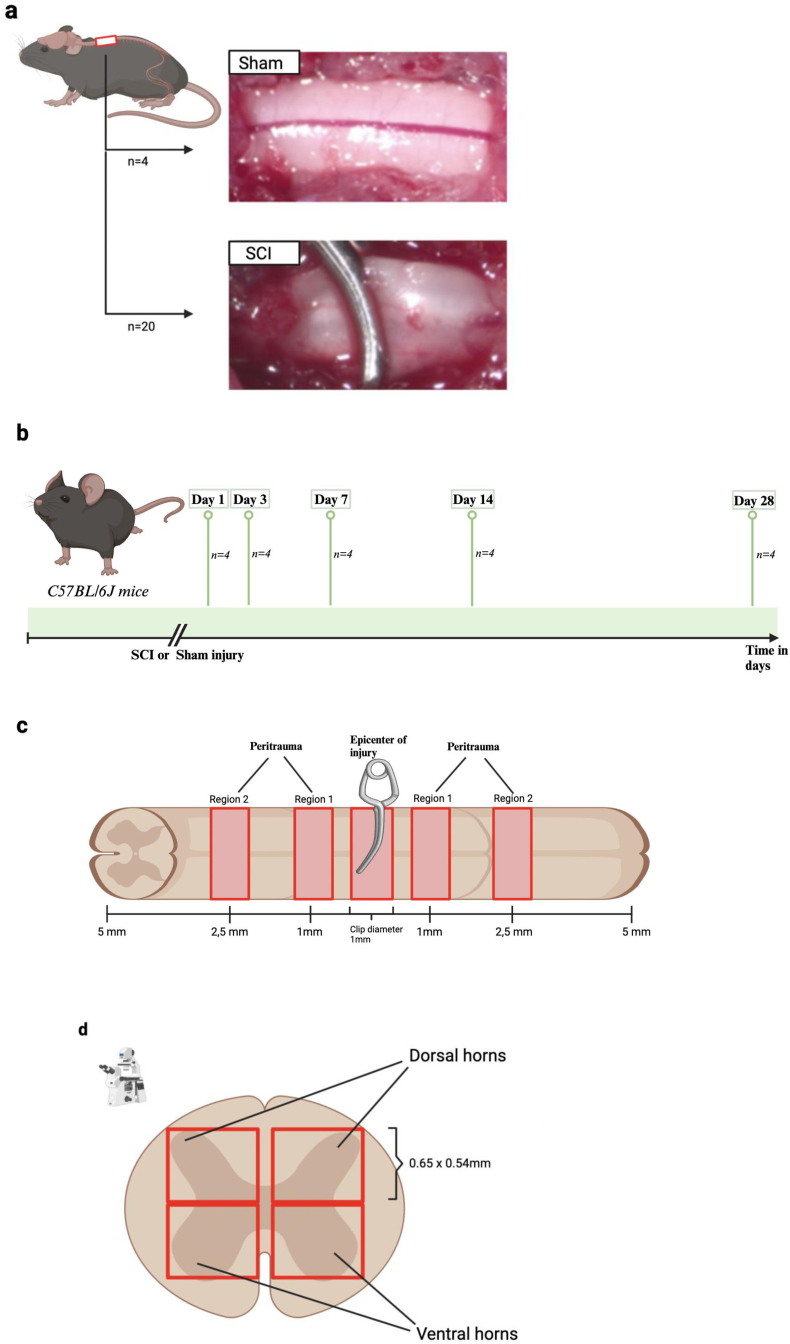
**Experimental setup: Temporospatial analysis of vascular injury and regeneration after SCI.** a) displays the intraoperative microscopic view of the murine spinal cord. The upper image on the right-hand side shows the spinal cord after a two-level laminectomy at Th6/7 of a sham animal (n = 4). In contrast, the image underneath displays the clip-compression model using a modified aneurysm clip that encloses the spinal cord of SCI animals circumferentially (n = 20, Fejota™). b) SC tissue of mice in the SCI group was analyzed at 5 different time points (days 1, 3, 7, 14, and 28; n = 4 per group). Before sacrifice, each animal (including Sham) received an intracardial FITC-Lectin perfusion, with circulation for 2min circulation followed by exsanguination. c) After sacrifice and organ explantation, a 1 cm part of the SC centered around the injury epicenter was sampled and immediately shock-frozen. Samples from 3 regions: epicenter (+- 0.5 mm), region 1 (+- 1 mm), and region 2 (+- 2.5 mm) were later cryosectioned, immunohistochemically processed, and analyzed. d) A schematic drawing of the spinal cord at an axial angle. The red squares indicate the areas of the ventral and dorsal horns that were assessed via immunofluorescence microscopy at a magnification of 20x (each area measuring around 0.65 mm (horizontal line) x 0.54 mm (vertical line) in size).

Animal experiments were approved by the local and governmental animal care committee (G0314/17) and the study is registered on the platform animaltestinfo.org (NTP-ID 00019011–1-7). For the induction of experimental SCI, a well-established murine clip-compression/contusion SCI model was used (Joshi and Fehlings, 2002a, 2002b). Adult female C57BL/6J mice (weight 20–24g; age 12–16 weeks, Charles River Laboratories) were randomly assigned to SCI (n = 20) or Sham (n = 4) injury (Fig. 1a). Within the SCI group, animals were randomly assigned to be sacrificed at defined time points after surgery (n = 4/timepoint): 1, 3, 7, 14, or 28 days (d, Fig. 1b). Before each procedure, animals were deeply anesthetized using i.p. ketamine (9 mg per 100g body weight) and xylazine (1 mg per 100g body weight). Mice were then fixated in a prone position and located under a surgical microscope. Following hair removal and disinfection, the skin was incised, the spinous processes identified, and the paravertebral muscles separated on both sides of the laminae. Each animal underwent a two-level laminectomy at Th6 and 7. Sham animals did not receive any further intervention at this point. In contrast, animals of the SCI group received a circumferential clip-compression at Th6/7 using a modified aneurysm clip (Fejota™) with a closing force of 5g for 60 s. Following NaCl 0,9% irrigation and hemostasis, the incision was closed in a layering fashion and animals received postoperative anesthesia with buprenorphin. Animals were left to recover in their respective cages under a heat lamp until awakening. Animal handling, preoperative/postoperative anesthesia, as well as surgery, were performed by the same experimenters to allow for minimum variation, and as previously described (Hubertus et al., 2024).

### 2.2 Tissue preparation and analysis

At days 1, 3, 7, 14, and 28 post-surgery, n = 4 animals per group (SCI) were deeply anesthetized and intracardially perfused with the *in vivo* fluorescent marker fluorescein isothiocyanate (FITC)-Lectin (2000 kDa 46946 Sigma Aldrich). Sham animals for CD31/FITC-Lectin and Ki-67 quantification were sacrificed after one (n = 1), 14 (n = 1) and 28 (n = 2) days, while for IgG-assessment after 7 days (n = 4) post sham surgery after receiving FITC-Lectin perfusion (see below). After allowing for a circulation time of 2 min following intracardial injection, the left ventricle was incised and animals were sacrificed through exsanguination. A 1 cm part of the SC centered around the injury epicenter was sampled and immediately shock-frozen using liquid nitrogen, then stored at −80 °C for later cryosectioning. Tissue blocks were fixated in a tissue-embedding medium and cryosectioned at a thickness of 10 μm through a microtome (Cryostat (HM560) Microm GmbH.) and stored at −80 °C for later immunohistochemical processing. For later analysis, three SC regions in each sample were predefined (Fig. 1c) and respective sections were allocated to those. Trauma epicenter (R0 = region 0) measured a width of 1 mm ( ± 0.5 mm), which represents the width of the modified aneurysm clip. Region 1 (R1) represented the area directly adjacent to R0 ( ± 1 mm), and region 2 (R2, ± 2.5 mm) included sections from tissue adjoining R1, at a farther distance from the injury epicenter. Because sham animals lacked an epicenter, a one cm section centered within the laminectomy region was taken.

### 2.3 Immunohistochemical processing and analysis

CD31 serves as an *ex vivo* marker for endothelial cells (EC), enabling the investigation of temporal and regional changes in the vasculature. To assess functional vessels (i.e. perfusion) *in vivo*, FITC-Lectin was administered intracardially prior to sacrifice. Given that FITC-Lectin circulates through the vasculature and binds specifically to the EC of blood vessels connected to the systemic circulation, the ratio of FITC-Lectin + to CD31^+^ signals was used, expressed as a percentage, to denote the perfusion ratio. To explore the temporal dynamics and regional distribution of vascular proliferation following SCI, EC proliferation was quantfied by counting the *ex vivo* nuclear proliferation marker Ki-67 in conjunction with CD31 and 4′,6-Diamidin-2-phenylindol (DAPI). Lastly, endogenous IgG (150 kDa) was stained to assess changes to the blood-spinal cord barrier (BSCB) following SCI along with anti-CD31 and TER-119, a marker for red blood cells (RBCs).

Quantification of cell count was performed using the software Cellprofiler™ (Version 4.2.5, Broadinstitute) (Carpenter et al., 2006; Stirling et al., 2021) for DAPI, CD31, FITC-Lectin, Ki-67, IgG& TER-119. FITC-Lectin + vessels were counted when they colocalized with CD31^+^ vessels and put in relation to the absolute CD31^+^ count in a given section. Ki-67+ signals were only counted when colocalization with DAPI (nuclear stain), and CD31 (vessels) was evident. Ki-67 counts of images were then normalized to the respective CD31 count, adjusting for different amounts of vessels within different sections. Next, the percentage (%) of IgG covering the total area of an image was quantified. Positive IgG counts that did not colocalize with CD31 or TER-119 were excluded, therefore excluding IgG from analysis within vessels and areas of hemorrhage.

### 2.4 Immunohistochemical staining

Glass slides with SC sections from respective areas were defrosted at room temperature (RT) for 5 min and then immersed in a 1:1 Acetone:Methanol solution at −20 °C for 10 min to achieve tissue permeabilization. Slides were then left to dry at RT, washed for 5 min in a Tris-Buffered Saline (TBS) solution, and blocked for 1h with a 10% Normal Donkey Serum (Dianova) at RT. Donkey serum was then removed and samples incubated with primary antibodies: CD31 (1:200 AF3628 R&D) and Ki-67 (1:50 RM-9106-S Thermo Scientific) or TER-119 (1:100 553671 BD Pharmingen) for 24h at 4 °C. Following primary incubation, glass slides were washed 3 × 5 min in TBS solution before incubating them with secondary antibodies (Alexa conjugated Dianova) for 2h at RT. Glass slides were then washed for 2 × 5min with a TBS solution and 2 × 5min in double distilled H2O (ddH2O) before using a glass cover medium with the nuclear stain DAPI (SCR-038448 Dianova) to fix the glass slide on the sample for microscopy.

During microscopy, images were taken using an Axio observer Z1 inverted immunofluorescence microscope (Carl Zeiss AG) and processed with the program Zeiss Zen 3.2 by an experimenter blinded to animal group, region, and timepoint. Images of the ventral and dorsal horns (four per section) were taken at 20x magnification (Fig. 1d).

### 2.5 Statistical analysis

Statistical consulting was provided by the Institute of Biometrics and Clinical Epidemiology (iBikE) of the Charité Universitätsmedizin for this study. All statistical analyses were performed using GraphPad Prism version 9 for Windows, GraphPad Software, Boston, Massachusetts USA (GraphPad Software, Inc.). Data sets were analyzed using mixed-effect models analysis for repeated measurements with the Dunnett multiple comparison test. To show trends over time we used simple linear regression analysis. Data are presented as mean ± standard deviation (SD). Differences were considered statistically significant at p ≤ 0.05.

## 3 Results & discussion

### 3.1 Vascular injury

**Fig. 2:**
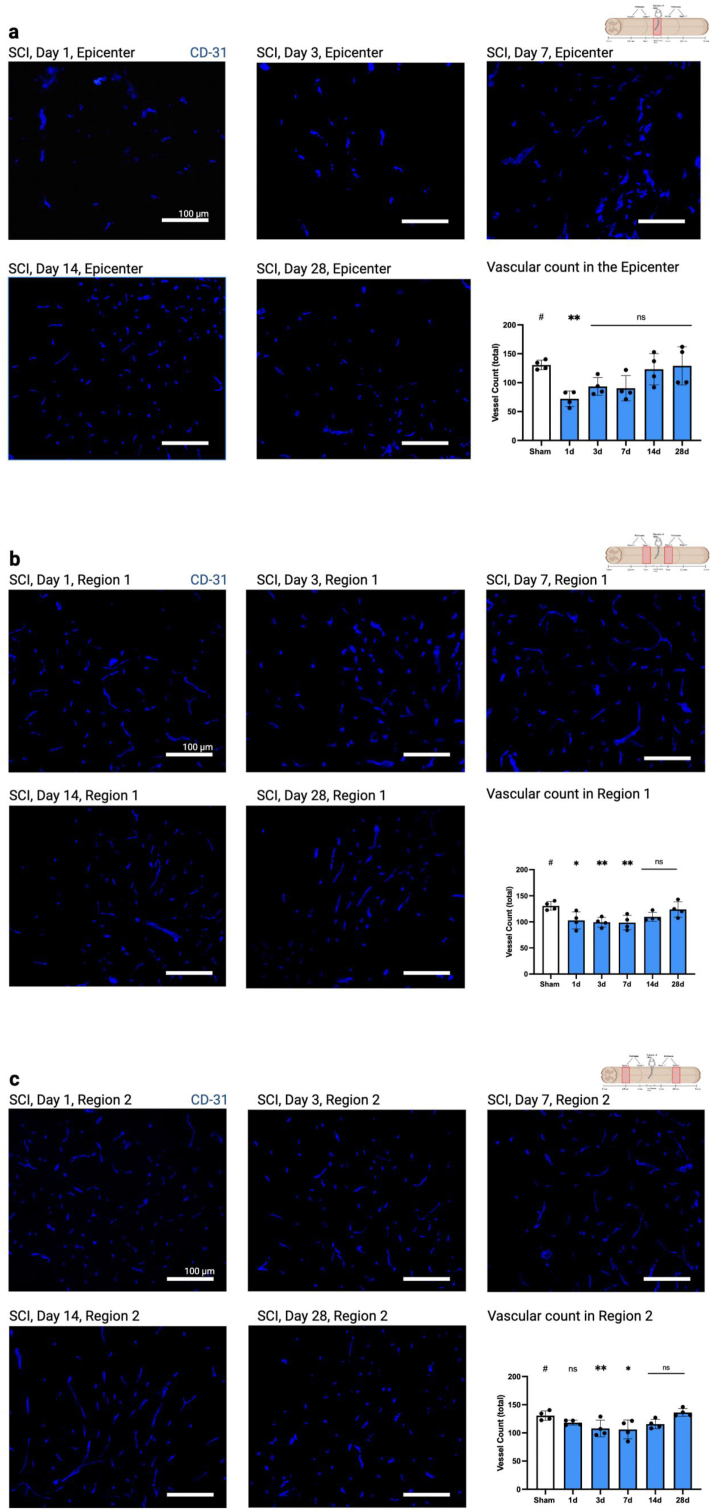
**Loss of CD31-positive vessels following SCI** a) epicenter of injury (R0): displayed is a drastic loss of CD31^+^ signals on the first day after SCI with a restitution occurring in the following days b) region 1 and c) region 2 show that vascular injury extends also beyond the epicenter and is not limited to day one, in fact in region 2 the loss of vasculature starts to become apparent only on day three. Scale bars, 100 μm. Vertical bars indicate mean ± SD. ∗P < 0.05, ∗∗P < 0.01.

When investigating the injury epicenter (R0) using total vascular count (CD31, Fig. 2a), an immediate decrease of around 45% (absolute count: 130.6 (sham) vs 72.19 (SCI), p = 0.005) in the vascular density at day one post-injury was observed (R0, Fig. 2b). Although total vascular count remained diminished over days three to 28, these reductions did not reach statistical significance (d3: p = 0.093; d7: p = 0.068; d14 and 28: p > 0.1). However, a clear upward trend between days one to 28 was evident through simple linear regression analysis (p = 0.039), indicating a gradual recovery of vessel count.

In the adjacent peritraumatic region R1, vascular counts on the first day after injury were also significantly reduced (p = 0.019). In contrast, in the more distant peritraumatic region R2, no significant differences were evident at any time point after injury (p = 0.076) (Fig. 2c-d). Further reductions in R1 and R2 were significant on days three (R1: p = 0.009; R2:p = 0.001) and seven (R1: p = 0.007 and p = 0.019) and returned to near Sham values by days 14 and 28 (p > 0.05).

**Fig. 3:**
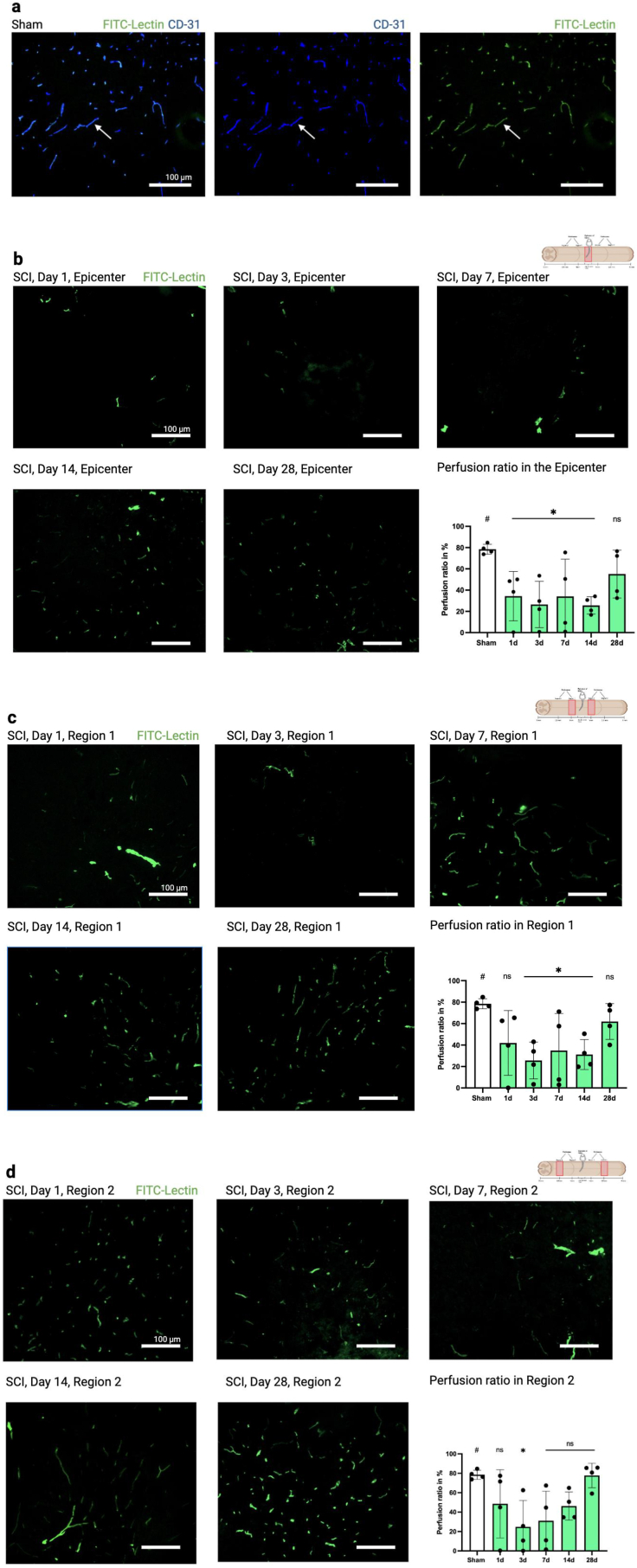
**Decrease in vessel perfusion following SCI.** a) The white arrow exemplarily points to the same CD31 and FITC-Lectin + vessel, which was counted for quantitative analysis. For illustration purposes colocalization images are not displayed in b-d, but only FITC-Lectin in green. b) In the epicenter starting from day one there is a significant decrease of perfusion, with a return to baseline occurring only on day 28 (p = 0.435). c) In region 1 a significant decrease of perfusion occurs on days three to 14, with a gradual return to baseline on day 28 (p = 0.73). d) In region 2 the most severe loss of perfusion occurs on day 3 after SCI, but a return to baseline occurs earlier at day 7 (p = 0.072). Scale bars, 100 μm. Vertical bars indicate mean ± SD. ∗P < 0.05, ∗∗P < 0.01.

To assess the perfusion ratio of these CD31^+^ vessels, we injected FITC-Lectin before sacrifice (Fig. 3a). In the injury epicenter, a significant decrease in vessel perfusion was observed from day one to day 14, with values approaching those of the sham group by day 28 (Fig. 3b), indicating a gradual recovery. In regions R1 and 2, the perfusion ratio was significantly reduced from days three to 14 in R1, however not on day one (R1: p = 0.111 and R2: p = 0.285, respectively). In R2, a significant reduction was only observed on day three (p = 0.032, Fig. 3c and d).

### 3.2 Vascular proliferation

**Fig. 4:**
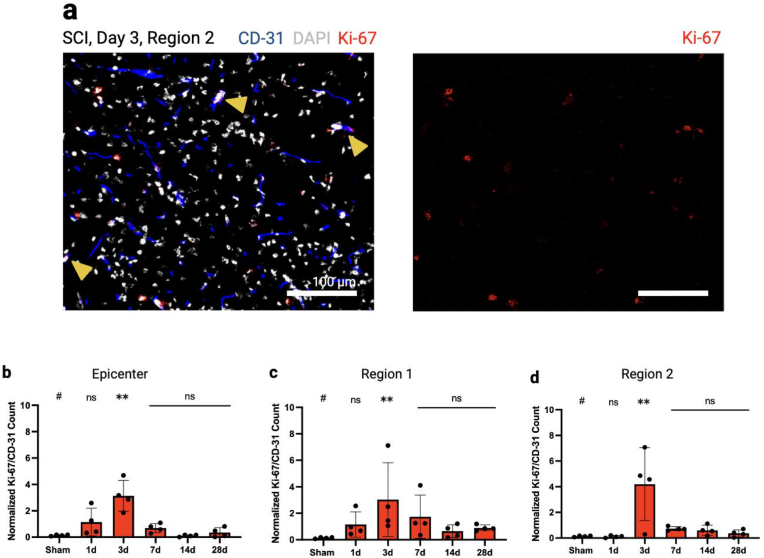
**Vascular proliferation following SCI**. a) Ki-67 (red) was counted when colocalized with CD31 (blue) and DAPI (white) positive signals. The yellow arrowheads exemplary point out to proliferating vessels. b-d) Ki-67 was significantly expressed throughout the spinal cord on day three, with the highest proliferation observed in region 2. Scale bars, 100 μm. Vertical bars indicate mean ± SD. ∗P < 0.05, ∗∗P < 0.01.

Next, we investigated vascular proliferation using Ki-67 as a marker of cell proliferation (see methods, Fig. 4a). On day three post-injury, a significant increase in proliferation was observed across all examined regions (R0: p < 0.01, R1: p = 0.0354, R2: p < 0.01, see Fig. 4b–d). At other time points, vascular proliferation did not reach statistical significance. Although the mean proliferation appeared to be highest in R2 (R0: 3.14; R1: 3.02; R2: 4.2), these regional differences were not statistically significant (e.g. p = 0.106 for R1 vs. R2).

### 3.3 Disruption of the blood-spinal-cord barrier

**Fig. 5:**
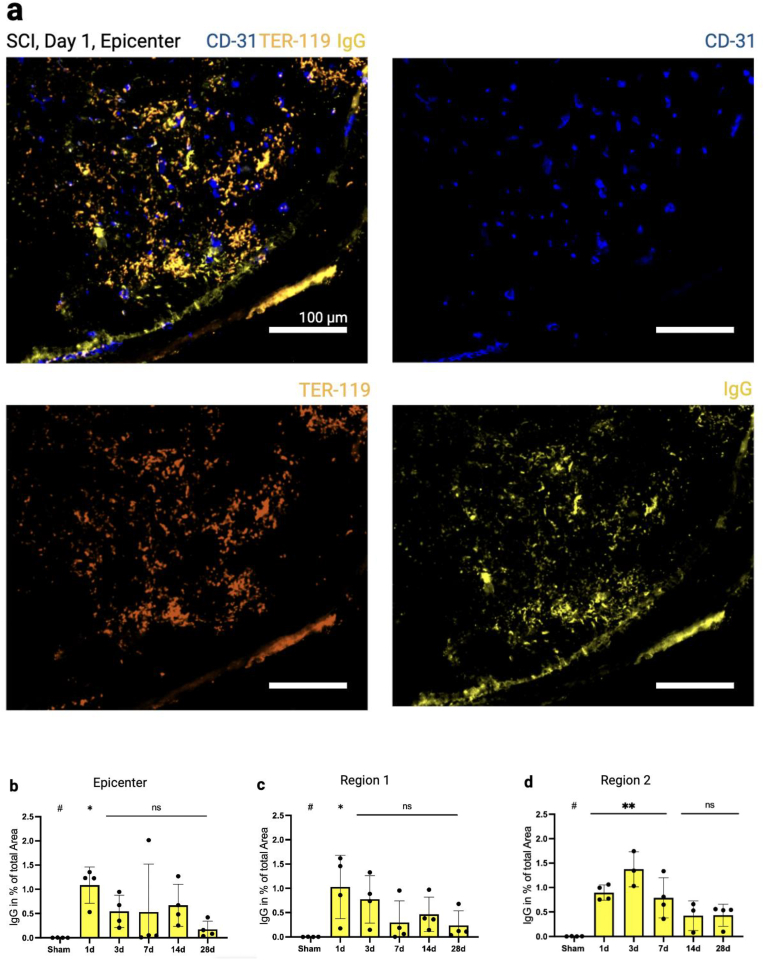
**Disruption of the Blood-Spinal Cord Barrier.** (a) Endogenous IgG (yellow) that did not colocalize with TER-119 (a marker for red blood cells, here in orange) or CD31 (blue, a marker for endothelial cells) was quantified. The majority of IgG is colocalizing with CD31 and TER-119 due to its association with the circulatory system. The peak of IgG extravasation occurs on day 1 in the epicenter (p = 0.011) and region 1 (p = 0.013) (b–c) and day 3 in region 2 (p < 0.001) (d). Scale bars, 100 μm. Vertical bars indicate mean ± SD. ∗P < 0.05, ∗∗P < 0.01.

A significant increase of extravascular immunoglobulin G (IgG, Fig. 5a) was detected as early as day one post-injury across all regions examined (R0: 1.01%, p = 0.01; R1: 1.02% p = 0.012; R2: 0,89%. p = 0.001) (Fig. 5b–d). In regions R0 and R1, the degree of extravascular IgG was highest on day one and decreased thereafter. In contrast, peak extravasation in R2 was observed on day three (1.3%, p < 0.001) and remained significantly elevated until day 14.

## 4 Discussion

In this study, we revealed the temporospatial dynamics of vascular changes following experimental SCI in an established murine clip-compression model over a period of 28 days and across multiple SC regions within and outside of the epicenter of primary injury. While we could demonstrate significant vascular loss and disruption of the BSCB within the epicenter, we showed that these changes occur in a time-delayed fashion also the adjacent peritraumatic regions. Ultimately, these changes induce a vascular proliferation that is not limited to the epicenter but also extends into the adjacent SC regions. While previous studies have focused predominantly on the epicenter of injury, this study provides important insights into the vascular dynamics occurring in adjacent and more distant perilesional areas. Our findings suggest that effective therapies should not only target the immediate injury site but also address these surrounding regions to better support overall SC recovery.

Vascular loss in the epicenter is attributed to the mechanical impact of the primary injury causing cell death of EC in the first few hours post-injury (Tator, 1991, 1995; Tator and Fehlings, 1991; Mautes et al., 2000; Figley et al., 2014a; Casella et al., 2006). Our data suggest a decrease in vasculature in the peritraumatic regions as well. This may result from secondary injury mechanisms, such as ischemia leading to EC death in peripheral areas (Tator, 1991, 1995; Mautes et al., 2000). Alternatively, the reduction might reflect a “dilutional” effect due to interstitial edema of the SC, which was not accounted for in this study (Griffiths, 1980; Griffiths and McCulloch, 1983; Oudega, 2012). A limitation of using CD31 as a vascular marker is that it does not provide information about the functional status of vessels at the time of sacrifice. Counts may include remnants of injured vessels, immature vessels, or vessels disconnected from circulation (Tator, 1991, 1995; Tator and Fehlings, 1991).

To gain more insights into the functional status of vessels, we performed intracardial injections of *in vivo* marker FITC-Lection prior to sacrifice. Unlike CD31, FITC-Lectin visualizes functionally perfused vessels, as it does not reach vessels disconnected from the circulatory system - such as those affected by microthrombosis, vasospasm, or newly forming vessels not yet integrated into the circulatory system (Tator and Fehlings, 1991; Figley et al., 2014a; Wallace et al., 1986; Koyanagi et al., 1993). Consequently, the ratio of FITC-Lectin + signals to CD31^+^ signals provides a perfusion ratio, which may be a more relevant marker of vascular integrity. We found that the degree of injury, as indicated by the perfusion ratio, was more extensive than suggested by CD31 counts alone. Our findings align with previous research indicating that the loss of perfusion is most severe at the injury epicenter (Figley et al., 2014a). We demonstrate that the peritraumatic regions also experience a significant perfusion loss, with areas closer to the epicenter being more severely affected. Unlike the epicenter, the peritraumatic regions exhibit a delayed perfusion loss, which is most pronounced on day three. Vascular dysfunction has been long recognized as a contributor to neurological deficits through an ongoing cascade of ischemia and cell death (Mautes et al., 2000; Roolfs et al., 2022). Beyond exacerbating the damage from the primary injury, the lack of functional blood vessels hinders the SC's ability to regenerate by failing to create the necessary microenvironment for recovery. Previously, we demonstrated that vascular regeneration is paralleled by partial recovery of functional outcomes in mice, highlighting the association between vascular repair and neural regeneration (Hubertus et al., 2024). Other studies have similarly shown that vascular regeneration intertwines with axonal regrowth and is associated with improved functional outcomes (Bearden and Segal, 2004; Ohab et al., 2006; Casella et al., 2002) and is associated with improved functional outcomes (Hubertus et al., 2024; Xiong et al., 2008). Consequently, it might be promising for future therapies targeting the SC microvasculature to not only be locally confined to the injury epicenter but to also involve peritraumatic regions. Focusing on the early restoration of local blood vessels and reestablishment of microvascular perfusion might hold the potential to enhance functional outcomes in patients with spinal cord injuries (Roolfs et al., 2022; Fassbender et al., 2011; Xiong et al., 2008, 2010). Particularly in peritraumatic regions, where perfusion loss occurs with a timely delay, there might be an opportunity to protect SC tissue at risk that is not initially damaged.

Our data on vascular proliferation suggests a robust proliferative response in the areas surrounding the primary injury site, potentially contributing to regeneration of spared neural tissues and thus improved recovery outcomes (Casella et al., 2002; Facchiano et al., 2002). Our findings are consistent with previous studies that vascular proliferation occurs within the first week after SCI and is pronounced in peritraumatic regions (Roolfs et al., 2022; Figley et al., 2014a; Loy et al., 2002; Casella et al., 2002, 2006; Whetstone et al., 2003). Endogenous angiogenesis is a tightly regulated process influenced by the tissue microenvironment (Chen et al., 2009). Notably, this period of maximum proliferation in the peritraumatic regions coincides with the lowest perfusion, suggesting that ischemia and hypoxia are key in promoting angiogenesis (Chen et al., 2009; Carmeliet and Jain, 2000). The synchronization of a peak in proliferation and reduced perfusion highlights the body's adaptive response to restore blood flow to ischemic tissue. Ischemia-induced hypoxia can upregulate angiogenic factors, promoting the formation of new blood vessels (Chen et al., 2009; Carmeliet and Jain, 2000). Understanding this temporal relationship underscores the importance of timely therapeutic interventions aimed at enhancing endogenous angiogenic responses.

The BSCB plays a critical role in the progression of secondary injury and resultant SC damage. Unlike previous studies, we utilized endogenous IgG to demonstrate early and severe BSCB damage. IgG, a protein with a molecular mass of approximately 150 kDa, is typically confined within the SC vasculature and rarely found outside those vessels. After SCI, however, the permeability of the BSCB increases dramatically, allowing IgG, like other proteins, and inflammatory cells to infiltrate the SC parenchyma. Our findings indicate that barrier disruption indicates a severe and early compromise of the BSCB not only at the injury epicenter but also extends to perilesional regions in a delayed fashion. Notably, peak disruption in the most distant region (R2) coincides with the period of maximum vascular proliferation (Fig. 4, Fig. 5d), suggesting that endogenous angiogenesis may contribute to increased vascular permeability, as previously indicated (Whetstone et al., 2003). Understanding the dynamics of BSCB disruption can inform therapeutic strategies aimed at preserving barrier integrity and mitigating secondary injury cascades. Previous studies have employed tracers of varying molecular sizes, ranging from less than 1 kDa to over 60 kDa, including C14-aminoisobutyric acid (Popovich et al., 1996), gadolinium (Cohen et al., 2009; Bilgen et al., 2001), horseradish peroxidase (Noble and Wrathall, 1987, 1988, 1989), and Evans Blue (Hubertus et al., 2024; Figley et al., 2014a). Consistent with other research, this study indicates that BSCB damage is most pronounced at the epicenter shortly after injury, with recovery occurring over subsequent days to weeks in a molecular size-dependent manner. For instance, Figley et al. reported significant Evans Blue (60 kDa) extravasation up to five days post-injury (Figley et al., 2014a), while the findings of Popovich and Cohen (Popovich et al., 1996; Cohen et al., 2009) using tracers smaller than 1 kDa suggest, that the BSCB may be compromised weeks to months post-injury. Our study's findings are limited by the use of IgG as a marker, given its relatively large size, which may not capture the nuances of permeability to smaller substances in the more chronic stages after SCI. While staining endogenous IgG can serve as an indicator of vascular permeability, it has several limitations. Unlike *in vivo* tracers applied at the time of sacrifice, endogenous IgG accumulation reflects permeability over a period rather than a specific time point. Consequently, this method indicates when the barrier becomes permeable to larger molecules like IgG, but does not provide detailed information about the dynamics of IgG in the extravascular space. Additionally, IgG detected on subsequent days may partially reflect accumulation from previous leakage events. Because IgG is a relatively large molecule, our data may overestimate barrier closure compared to studies using smaller tracers, as has been demonstrated in other research (Popovich et al., 1996; Cohen et al., 2009).

Our study has limitations that warrant consideration. Quantifying perfusion via FITC-Lectin showed high variability observed within some animals, likely due to a combination of biological and technical factors. Despite standardized procedures (e.g., maintaining a consistent closing pressure of 5g and using the same surgeon for all animals), individual animals may have sustained more extensive SCI damage. Additionally, FITC-Lectin-a fluorescent marker that predominantly labels smaller vessels-may yield lower perfusion values under suboptimal systemic circulation. Supporting this possibility, CD31 staining and other vascular metrics did not exhibit the same level of inter-animal variability, suggesting that the observed discrepancies may be method-related rather than due to intrinsic differences in vascular density. Further, Sex has been identified as a biological factor that can influence results of preclinical animal studies including SCI (Miller et al., 2017; Stewart et al., 2020). Emerging research highlights that sex-specific differences in inflammatory responses, hormonal regulation, and recovery profiles can influence SCI outcome (Miller et al., 2017; Datto et al., 2015). Using only female mice limits the generalizability of results, therefore future studies should include both male and female subjects to provide a more comprehensive understanding of sex-dependent effects on vascular regeneration and recovery after SCI. Lastly, while we provide extensive temporal and spatial data on vascular permeability and proliferation using immunohistochemistry, we do not delve into the underlying molecular mechanisms driving these vascular changes. Advanced techniques, such as single-cell RNA sequencing or proteomic analyses, would help to elucidate the signaling pathways and cellular interactions involved in vascular injury and repair that are specific in peritraumatic regions and at different posttraumatic timepoints (Milich et al., 2021). Understanding these molecular interactions will furthermore be essential for the development of targeted therapeutic strategies to enhance vascular repair and improve outcomes after SCI.

## 5 Conclusion

This study provides comprehensive immunohistochemical insight into the temporospatial dynamics of vascular injury and regeneration following SCI, utilizing an established murine clip-compression model. By characterizing vascular changes in three distinct SC regions - the injury epicenter and two adjacent peritraumatic areas with mounting distance from the injury epicenter - across various time points (days 1, 3, 7, 14, 28), a temporospatial map of vascular injury and regeneration is given.

Our findings reveal significant early vascular disruption at the injury epicenter, with gradual recovery observed over the course of 28 days. The peritraumatic regions exhibit a delayed vascular loss, indicative of secondary injury mechanisms extending beyond the initial trauma site in a delayed fashion. Vascular proliferation peaks after three days, particularly in the distal peritraumatic region (R2), suggesting a robust endogenous regenerative response aimed at restoring vascular integrity within regions adjacent to the initial injury site. Additionally, an early compromise of the BSCB is observed, with IgG extravasation peaking on the first day after injury at the epicenter and after three days in distant regions, displaying a delayed spreading of this secondary injury mechanism along the injury site.

These results underscore the critical role of vascular dynamics - including vascular loss, proliferation, and barrier integrity - in the pathophysiological and regenerational processes following SCI. The temporospatial mapping of vascular injury and regeneration provided by this study offers valuable insights for the future development of targeted therapeutic interventions. Specifically, our findings highlight the importance of early and continuous interventions aimed at stabilizing the BSCB and promoting revascularization not only at the injury epicenter but also in the surrounding peritraumatic regions.

Future research is necessary to elucidate the molecular mechanisms underlying these vascular responses. Understanding these pathways will be pivotal in optimizing therapeutic strategies including pharmacological agents or cell-based therapies, to enhance vascular repair and thus improve neurological recovery after SCI. In particular gene therapy approaches hold particular promise in enhancing vascular repair and neuroprotection by promoting the expression of factors like VEGF-A, which support angiogenesis and tissue recovery. Such therapies, by leveraging endogenous repair mechanisms, could complement conventional treatments to improve outcomes in SCI (Figley et al., 2014b). Ultimately, such advancements could contribute to the development of effective treatments that mitigate secondary injury cascades and promote functional restoration in patients suffering from SCI.

## Ethical vote

Animal experiments were approved by the local and governmental animal care committee (G0314/17) and the study is registered on the platform animaltestinfo.org (NTP-ID 00019011–1-7).

## Author contributions

VH, PV, and CE designed the study concept. VH performed the animal surgeries. VH, LW and LM performed animal perfusion and the majority of cryosectioning. CE performed the immunohistochemical staining, image analysis as well as statistical analysis. CE performed the literature review, created figures and wrote the draft of the manuscript. VH, PV and CE revised the manuscript crucially. All authors listed participated in the revision of the manuscript.

## Financing, funding and appreciation

This study was financed by the 2019 INTEGRA EANS research fund. VH is funded by the Berlin Institute of Health (BIH) Charité Clinician Scientist Program. LR is funded by Deutsche Studienstiftung.

## Conflicts of interest

No conflicts of interest exist regarding the submission and publication of this manuscript. All authors have read the manuscript and agreed to its submission for publication.

## Abbreviations

ANOVA: Analysis of Variance
BSCB: Blood-Spinal Cord Barrier
CNS: Central Nervous System
EC: endothelial cells
FITC: Fluorescein Isothiocyanate
Gd: Gadolinium
IgG: Immunoglobulin G
i.p.: Intraperitoneal
MRI: Magnetic Resonance Imaging
RBC: Red Blood Cells
RT: Room Temperature
SC: Spinal Cord
SCI: Spinal Cord Injury
SD: Standard Deviation
TBS: Tris-Buffered Saline

## References

[bib1] Ahuja C.S., Wilson J.R., Nori S., Kotter M.R.N., Druschel C., Curt A. (2017). Traumatic spinal cord injury. Nat. Rev. Dis. Prim..

[bib2] Ahuja C.S., Nori S., Tetreault L., Wilson J., Kwon B., Harrop J. (2017). Traumatic spinal cord injury-repair and regeneration. Neurosurgery.

[bib3] Bearden S.E., Segal S.S. (2004). Microvessels promote motor nerve survival and regeneration through local VEGF release following ectopic reattachment. Microcirculation.

[bib4] Bilgen M., Abbe R., Narayana P.A. (2001). Dynamic contrast-enhanced MRI of experimental spinal cord injury: in vivo serial studies. Magn. Reson. Med..

[bib5] Carmeliet P., Jain R.K. (2000). Angiogenesis in cancer and other diseases. Nature.

[bib6] Carpenter A.E., Jones T.R., Lamprecht M.R., Clarke C., Kang I.H., Friman O. (2006). CellProfiler: image analysis software for identifying and quantifying cell phenotypes. Genome Biol..

[bib7] Casella G.T., Marcillo A., Bunge M.B., Wood P.M. (2002). New vascular tissue rapidly replaces neural parenchyma and vessels destroyed by a contusion injury to the rat spinal cord. Exp. Neurol..

[bib8] Casella G.T., Bunge M.B., Wood P.M. (2006). Endothelial cell loss is not a major cause of neuronal and glial cell death following contusion injury of the spinal cord. Exp. Neurol..

[bib9] Chen L., Endler A., Shibasaki F. (2009). Hypoxia and angiogenesis: regulation of hypoxia-inducible factors via novel binding factors. Exp. Mol. Med..

[bib10] Cohen D.M., Patel C.B., Ahobila-Vajjula P., Sundberg L.M., Chacko T., Liu S.J. (2009). Blood-spinal cord barrier permeability in experimental spinal cord injury: dynamic contrast-enhanced MRI. NMR Biomed..

[bib11] Datto J.P., Bastidas J.C., Miller N.L., Shah A.K., Arheart K.L., Marcillo A.E. (2015). Female rats demonstrate improved locomotor recovery and greater preservation of white and gray matter after traumatic spinal cord injury compared to males. J. Neurotrauma.

[bib12] Ding W., Hu S., Wang P., Kang H., Peng R., Dong Y. (2022). Spinal cord injury: the global incidence, prevalence, and disability from the global burden of disease study 2019. Spine.

[bib13] Facchiano F., Fernandez E., Mancarella S., Maira G., Miscusi M., D'Arcangelo D. (2002). Promotion of regeneration of corticospinal tract axons in rats with recombinant vascular endothelial growth factor alone and combined with adenovirus coding for this factor. J. Neurosurg..

[bib14] Fassbender J.M., Whittemore S.R., Hagg T. (2011). Targeting microvasculature for neuroprotection after. SCI Neurotherapeut..

[bib15] Figley S.A., Khosravi R., Legasto J.M., Tseng Y.F., Fehlings M.G. (2014). Characterization of vascular disruption and blood-spinal cord barrier permeability following traumatic spinal cord injury. J. Neurotrauma.

[bib16] Figley S.A., Liu Y., Karadimas S.K., Satkunendrarajah K., Fettes P., Spratt S.K. (2014). Delayed administration of a bio-engineered zinc-finger VEGF-A gene therapy is neuroprotective and attenuates allodynia following traumatic spinal cord injury. PLoS One.

[bib17] Safdarian Mahdi, Trinka Eugen, Rahimi-Movaghar Vafa, Thomschewski Aljoscha, Amirali Aali, Abady Gdiom Gebreheat, Abate Semagn Mekonnen (2023). Global, regional, and national burden of spinal cord injury, 1990-2019: a systematic analysis for the Global Burden of Disease Study 2019. Lancet Neurol..

[bib18] Griffiths I.R. (1980). Trauma of the spinal cord. Vet. Clin. North Am. Small Anim. Pract..

[bib19] Griffiths I.R., McCulloch M.C. (1983). Nerve fibres in spinal cord impact injuries. Part 1. Changes in the myelin sheath during the initial 5 weeks. J. Neurol. Sci..

[bib20] Hubertus V., Meyer L., Waldmann L., Roolfs L., Taheri N., Kersting K. (2024). Identification of a therapeutic window for neurovascular unit repair after experimental spinal cord injury. J. Neurotrauma.

[bib21] Joshi M., Fehlings M.G. (2002). Development and characterization of a novel, graded model of clip compressive spinal cord injury in the mouse: Part 1. Clip design, behavioral outcomes, and histopathology. J. Neurotrauma.

[bib22] Joshi M., Fehlings M.G. (2002). Development and characterization of a novel, graded model of clip compressive spinal cord injury in the mouse: Part 2. Quantitative neuroanatomical assessment and analysis of the relationships between axonal tracts, residual tissue, and locomotor recovery. J. Neurotrauma.

[bib23] Koyanagi I., Tator C.H., Theriault E. (1993). Silicone rubber microangiography of acute spinal cord injury in the rat. Neurosurgery.

[bib24] Kumar R., Lim J., Mekary R.A., Rattani A., Dewan M.C., Sharif S.Y. (2018). Traumatic spinal injury: global Epidemiology and worldwide volume. World Neurosurg..

[bib25] Kwon B.K., Tetreault L.A., Evaniew N., Skelly A.C., Fehlings M.G. (2024). AO spine/praxis clinical practice guidelines for the management of acute spinal cord injury: an introduction to a focus issue. Global Spine J..

[bib26] Loy D.N., Crawford C.H., Darnall J.B., Burke D.A., Onifer S.M., Whittemore S.R. (2002). Temporal progression of angiogenesis and basal lamina deposition after contusive spinal cord injury in the adult rat. J. Comp. Neurol..

[bib27] Mautes A.E., Weinzierl M.R., Donovan F., Noble L.J. (2000). Vascular events after spinal cord injury: contribution to secondary pathogenesis. Phys. Ther..

[bib28] Milich L.M., Choi J.S., Ryan C., Cerqueira S.R., Benavides S., Yahn S.L. (2021). Single-cell analysis of the cellular heterogeneity and interactions in the injured mouse spinal cord. J. Exp. Med..

[bib29] Miller L.R., Marks C., Becker J.B., Hurn P.D., Chen W.J., Woodruff T. (2017). Considering sex as a biological variable in preclinical research. Faseb. J..

[bib30] Ng M.T., Stammers A.T., Kwon B.K. (2011). Vascular disruption and the role of angiogenic proteins after spinal cord injury. Transl. Stroke Res..

[bib31] Noble L.J., Wrathall J.R. (1987). The blood-spinal cord barrier after injury: pattern of vascular events proximal and distal to a transection in the rat. Brain Res..

[bib32] Noble L.J., Wrathall J.R. (1988). Blood-spinal cord barrier disruption proximal to a spinal cord transection in the rat: time course and pathways associated with protein leakage. Exp. Neurol..

[bib33] Noble L.J., Wrathall J.R. (1989). Distribution and time course of protein extravasation in the rat spinal cord after contusive injury. Brain Res..

[bib34] Ohab J.J., Fleming S., Blesch A., Carmichael S.T. (2006). A neurovascular niche for neurogenesis after stroke. J. Neurosci..

[bib35] Oudega M. (2012). Molecular and cellular mechanisms underlying the role of blood vessels in spinal cord injury and repair. Cell Tissue Res..

[bib36] Paraplegiologie DMGf. Pressemappe Querschnittlähmung.

[bib37] Popovich P.G., Horner P.J., Mullin B.B., Stokes B.T. (1996). A quantitative spatial analysis of the blood-spinal cord barrier. I. Permeability changes after experimental spinal contusion injury. Exp. Neurol..

[bib38] Roolfs L., Hubertus V., Spinnen J., Shopperly L.K., Fehlings M.G., Vajkoczy P. (2022). Therapeutic approaches targeting vascular repair after experimental spinal cord injury: a systematic review of the literature. Neurospine.

[bib39] Rupp R., Jersch P., Schuld C., Schweidler J., Benning N.H., Knaup-Gregori P. (2021). [Germany-wide, web-based ParaReg registry for lifelong monitoring of people with spinal cord injury: data model, ethico-legal prerequisites and technical implementation]. Gesundheitswesen.

[bib40] Stewart A.N., MacLean S.M., Stromberg A.J., Whelan J.P., Bailey W.M., Gensel J.C. (2020). Considerations for studying sex as a biological variable in spinal cord injury. Front. Neurol..

[bib41] Stirling D.R., Swain-Bowden M.J., Lucas A.M., Carpenter A.E., Cimini B.A., Goodman A. (2021). CellProfiler 4: improvements in speed, utility and usability. BMC Bioinf..

[bib42] Tator C.H. (1991). Review of experimental spinal cord injury with emphasis on the local and systemic circulatory effects. Neurochirurgie.

[bib43] Tator C.H. (1995). Update on the pathophysiology and pathology of acute spinal cord injury. Brain Pathol..

[bib44] Tator C.H., Fehlings M.G. (1991). Review of the secondary injury theory of acute spinal cord trauma with emphasis on vascular mechanisms. J. Neurosurg..

[bib45] Wallace M.C., Tator C.H., Frazee P. (1986). Relationship between posttraumatic ischemia and hemorrhage in the injured rat spinal cord as shown by colloidal carbon angiography. Neurosurgery.

[bib46] Whetstone W.D., Hsu J.Y., Eisenberg M., Werb Z., Noble-Haeusslein L.J. (2003). Blood-spinal cord barrier after spinal cord injury: relation to revascularization and wound healing. J. Neurosci. Res..

[bib47] WHO (2024). Spinal cord injury.

[bib48] Xiong Y., Lu D., Qu C., Goussev A., Schallert T., Mahmood A. (2008). Effects of erythropoietin on reducing brain damage and improving functional outcome after traumatic brain injury in mice. J. Neurosurg..

[bib49] Xiong Y., Mahmood A., Chopp M. (2010). Neurorestorative treatments for traumatic brain injury. Discov. Med..

